# Holographic tomography of the diatom *Skeletonema pseudocostatum* used as a bioindicator of heavy metal-polluted waters

**DOI:** 10.1371/journal.pone.0322960

**Published:** 2025-05-08

**Authors:** Maria Antonietta Ferrara, Elena Cavalletti, Vittorio Bianco, Lisa Miccio, Giuseppe Coppola, Pietro Ferraro, Angela Sardo

**Affiliations:** 1 Institute of Applied Sciences and Intelligent Systems, Unit of Naples, Italian National Research Council (ISASI-CNR), Via Pietro Castellino 111, Naples, Italy; 2 Department of Ecosustainable Marine Biotechnology, Stazione Zoologica Anton Dohrn, Naples, Italy; 3 Institute of Applied Sciences and Intelligent Systems, Italian National Research Council (ISASI-CNR), Pozzuoli (Naples), Italy; Jinan University, CHINA

## Abstract

Heavy metal contamination in aquatic environments poses a significant threat to microbial communities, yet the subcellular responses of phytoplankton to metal stress remain poorly understood. In particular, the effects of heavy metal exposure on the structural and physiological properties of diatoms require further investigation. Here, we analyze the impact of cadmium (Cd) and copper (Cu) exposure on the subcellular structures of the diatom *Skeletonema pseudocostatum* using holographic tomography. This imaging technique enables detailed visualization and quantitative analysis of diatom subcomponents, including frustules, protoplasm, vacuoles, and chloroplasts, under varying metal concentrations. The study aims to understand the changes in the mean refractive index (RI) and concentration (e.g., the ratio among cell dry mass and its biovolume) as indicators of cellular response to metal stress and to infer if such diatom can be used as sentinel species of heavy metal pollution. Findings indicate that diatoms exhibit significant variations in RI and internal cell density when exposed to different metal concentrations. Lower RI values observed at higher metal concentrations, can be considered as a sign of stress due to cytoplasm extrusion and/or vacuolization. The results highlight the potential of using *S. pseudocostatum* as a bioindicator for monitoring water metal pollution. Moreover, the results show that holographic tomography as useful tool for non-invasive, high-resolution cellular imaging of phytoplankton in environmental studies.

## Introduction

Water pollution is a pressing global issue with far-reaching consequences for both human and environmental health. As human activities continue to release pollutants into our water bodies, the need for fast and effective methods of monitoring water quality becomes increasingly important. In this context, diatoms, a group of unicellular, photosynthetic algae, have gained significant attention as valuable bioindicators to assess the ecological health of aquatic ecosystems [1–3]. Their ability to reflect changes in water quality and their sensitivity to environmental stressors coupled with their ecological and economic significance, make them ideal candidates for assessing the impacts of human activities on water resources.

The use of diatoms as indicators of water pollution is based on the premise that different species of diatoms have varying tolerances to specific pollutants and environmental stressors, allowing researchers to pinpoint the types of contaminants present in an aquatic ecosystem and take steps to mitigate the impacts of pollution. For example, certain diatom species thrive in clean, nutrient-poor waters, while others are better adapted to cope with elevated levels of organic matter, heavy metals (HMs), or other contaminants [4–7]. Moreover, their abundance, diversity, and response to various pollutants as well as their diversity in tolerance and sensitivity make them valuable tools in assessing the water quality and ecological health of aquatic ecosystems. Monitoring the composition of diatom populations and studying their responses to pollutants can provide insights into the impacts of anthropogenic activities on water resources, help identify sources of pollution and identify areas that may be experiencing pollution.

Furthermore, the economic potential of diatoms has been highlighted, as they can be utilized for the production of added-value products, energy generation, and even as a source of pharmaceuticals and aquaculture feedstocks [8].

In addition to their role as indicators of water quality, diatoms also play a vital role in ecosystem functioning. As primary producers, they form the base of the aquatic food web and provide food for a wide range of organisms [9]. Changes in diatom populations can have cascading effects throughout the ecosystem, impacting species at higher trophic levels.

In contemporary research, novel techniques have been developed to better understand the health status and responses of diatom populations to environmental stressors. These techniques include advanced imaging methods such as fluorescence microscopy, which allows for the visualization of cellular structures and physiological processes within diatoms. Fluorescence-based assays can also be used to assess the physiological status of diatoms, including measures of photosynthetic activity and cell viability.

Furthermore, molecular techniques such as DNA sequencing and gene expression analysis are increasingly being used to study diatom communities and their responses to environmental changes [10–12]. These techniques enable researchers to identify diatom species present in a sample and assess their genetic responses to pollutants or other stressors.

In addition to these molecular and imaging techniques, traditional methods such as microscopy (optical and electron) and cell counting are still widely used to monitor diatom populations, to identify cell impairments and growth rate reduction related to water quality [13]. These methods provide valuable information about diatom abundance, species composition, and community structure, which can be used to infer the overall health of aquatic ecosystems. However, these techniques are often time-consuming, costly, and do not facilitate early detection of environmental pollution. Moreover, optical microscope observations may aid in identifying dead cells and significant alterations in algal morphology, so they typically only provide rough distinctions between healthy and unhealthy diatoms.

Overall, the integration of advanced imaging, molecular, and traditional techniques has greatly enhanced our ability to investigate the health and responses of diatom populations to environmental stressors. By combining these approaches, scientists can gain a comprehensive understanding of the factors influencing diatom ecology and use this knowledge to inform conservation and management efforts aimed at preserving water quality and ecosystem health.

Digital Holography (DH), first introduced by Gabor in 1948 [14], revolutionized microscopic imaging by enabling non-invasive, label-free, and high-resolution 3D imaging of transparent objects such as plankton and diatoms. This technique employs digital sensors to record holograms, which are then computationally reconstructed to reveal both amplitude and phase information about the sample [15,16]. Over the years, DH has evolved with advancements in computational algorithms, detector technology, and optical configurations. Digital in-line holography (DIH) has been widely used for tracking and identifying biological cells, providing insights into their dynamic behaviors [17].

As DH progressed, it paved the way for the development of Holotomography (HT), a state-of-the-art imaging technique that combines the principles of holography and tomography. HT enables the non-invasive, high-resolution, three-dimensional and quantitative imaging of biological samples, providing detailed insights into their intricate structures. This non-invasive technique uses coherent light to create multiple 2D holograms acquired at varying illumination angles, which are then computationally reconstructed to produce detailed 3D refractive index (RI) distributions of the sample, which can serve as a direct indicator of physiological stress responses, and allowing for the assessment of both morphological information and the quantification of dry mass and dry mass concentration [18,19].

DH has been extensively used to image phytoplankton and zooplankton [20], and to describe the wide heterogeneity of diatom species populating various aquatic environments [21]. Mostly, in-line holographic apparatus has been used relying on high-coherence laser sources or low-coherence light, e.g., Light Emitting Diodes (LEDs). With the aim to enable in situ monitoring of the diatom’s compositions and fluxes, field portable setups have been developed in the form of high-throughput RGB imaging flow cytometers [9,13]. *Ditylum brightwellii*, *Ceratium furca*, *Eucampia cornuta*, species belonging to the genera *Lauderia*, *Leptocylindrus*, *Lithodesmium*, *Pleurosigma*, *Dinophysis*, *Thalassionema* and zooplanktonic species such as Ciliates, are a few examples of strains identified using a holographic microscope operating in continuous flow [13]. Algal bloom phenomena are carefully monitored by holographic sensors. The spatial and temporal patterns of *Pseudo-nitzschia*, a toxic alga producing domoic acid, have been reported to guide fishery activities and foster public health [22]. Besides, submersible holographic microscopes have been successfully proposed to inspect the water column by imaging diatoms underwater [23,24]. All these previous studies suggest that the phase-contrast readout is suitable for describing the inter-species heterogeneity for diatom classification purposes [25,26]. The use of machine learning approaches applied to phase-contrast signatures has empowered and fostered these processes [27]. However, the integral phase-contrast is a 2D measure that may be not informative enough to describe the intrinsic intra-species variability of diatom populations and the changes of their inner traits induced by environmental and anthropic factors. HT is one of the most suitable methods to resolve sample features along the optical axis and to decouple the refractive index from the physical thickness.

Nevertheless, only a limited set of studies have attempted to map the 3D refractive index distribution of marine diatoms using HT [28–31]. *Coscinusdiscus* [30], *Cylindrotheca* [28], *Navicula*, *Pseudo-nitzschia* and *Thalassiosira* spp. [29,31] are good examples in this sense.

The genus *Skeletonema* has never been characterized in full using HT. The sole exception is a paper where it was used as a test sample to benchmark a method for in-flow HT, which was the very first attempt to bridge the gap between HT and flow cytometry [32].

*Skeletonema* spp. are characterized by cylindrical shape and form long chains, which are connected by external filaments [33,34]. This genus is widespread in the Gulf of Naples and its presence and abundance can indicate changes in water quality and nutrient levels, making them useful in monitoring environmental conditions and the health of aquatic ecosystems.

Recently, the sensitivity of *S. pseudocostatum* to copper doses has been investigated using 2D phase-contrast maps [3]. Results of this study spotlight the species *S. pseudocostatum* as a suitable bioindicator of metal-induced stress and encouraged us to perform a more in-depth analysis of potential modifications of their inner traits as a result of heavy metals exposure.

This paper aims to analyze the impact of heavy metal treatments on the subcellular parts of this diatom, focusing on changes in some parameters such as mean refractive index, average density, and volume. The study employs HT to identify the for the first time the subparts of the above-mentioned species (frustule, protoplasm, vacuole, and chloroplast), and to assess eventual impairments of the whole cells and their chloroplasts when exposed to different doses of heavy metals. By leveraging RI ranges for each diatom subparts, this research provides a dose-dependent analysis that reveals adaptive mechanisms in diatoms, suggesting their potential for use in real-time bioindication of water quality.

## Methods

### Culture conditions and sample preparation for HT

*S. pseudocostatum* (initial cell density: 60000 c/mL) was maintained in f/2 medium (salinity 36) [35,36] in 75-cm^2^ sterile flasks (working volume: 100 ml), provided by 0.22-µm filters on the cap to ensure the entrance of sterile air. Species were grown at 18 °C, an irradiance of ca. 150 µmol photons m^-2^ s^-1^, a 12:12 light: dark photoperiod. Cultures were exposed to 0 (control samples), 10 or 25 µ M of copper (supplied as CuSO_4_ ∙ 5H_2_O) and cadmium (supplied ad CdCl_2_) for three days. Each experiment was performed in triplicate. Cells were daily counted under an inverted microscope (Aviovert 200, Oberkochen, Germany) with a Bürker chamber (Mannhein, Germany). For HT acquisitions, 5 ml of each algal sample was collected at the start and every 24 hours in a 15-mL falcon tube and fixed with a Lugol iodine solution (30 µl/mL). Samples were stored at 4°C in the dark until analysis. 70- µ L aliquots of each sample were placed in TomoDish instead of conventional Petri dishes. TomoDish is a specialized optical glass-bottom dish designed to minimize optical aberrations and enhance the quality of three-dimensional quantitative phase imaging, ensuring optimal light transmission and accurate refractive index measurements.

### Holographic tomography

The tomographic characterization has been performed using the Tomocube® HT-2H microscope [37]. Tomocube is a tomographic imaging system that utilizes holography-based technology to provide 3D images of biological samples. This setup includes an optical system consisting of a laser, digital micromirror devices, and a high-resolution camera [38,39]. The system operates with a water immersion objective lens providing a magnification of 60× (numerical aperture NA = 1.2). The resolution of the system is approximately 110 nm laterally and 370 nm axially, enabling high-precision visualization of subcellular structures. The field of view (FOV) of the TomoCube HT-2H is approximately 80 × 80 µm, which allows the simultaneous imaging of multiple cells within a single frame. The acquisition time for a complete three-dimensional tomogram is less than one second (0.4 sec), with imaging speed of 150 fps for 2D holography and 2.5 fps for 3D holography, ensuring minimal phototoxicity and enabling real-time observation of dynamic cellular processes.

The principle of HT involves illuminating a sample with low-power visible light from different angles, recording the corresponding holograms using interferometry, measuring the phase shifts in the transmitted light reconstructing the diffracted optical field. These shifts are used to reconstruct a 3D image based on the refractive index (RI) of the sample, enabling detailed, label-free visualization and quantitative analysis of cellular structures. Basically, the holographic data are processed using advanced algorithms to reconstruct a high-resolution 3D image of the sample [30,38]. While the depth resolution could be a limitation, leading to elongation of the cell shape along the depth direction, the Tomocube HT-2H is highly reliable for statistical measurements, especially when analyzing large datasets of cells, minimizing errors through averaging multiple measurements, acquiring holograms from multiple angles, and using advanced algorithmic approaches to compensate for distortions [40]. Moreover, the errors associated with some distortion in measurements of volume, dry mass, and concentration are generally minimized thanks to the system’s ability to collect holographic data over a wide depth range, resulting in an accurate 3D representation of the cell [41].

The used HT microscope allows combining diffraction tomography with 3D fluorescence imaging to provide high-resolution, real-time images of live cells and tissues without the need for sample preparation. This setup offers several advantages over traditional microscopy techniques. In particular, its ability to visualize the 3D RI distribution of samples in their natural state without the need for sample preparation or staining, with high resolution imaging at sub-cellular levels, and real-time imaging capabilities are great advantages in study of diatom samples.

### Results and discussionGrowth of *S. pseudocostatum* exposed to Cu and Cd

**Fig 1:**
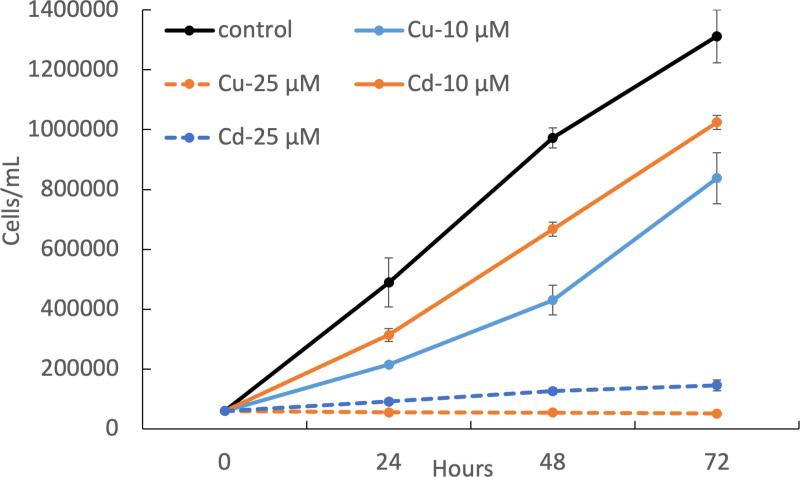
Growth cultures of *S. pseudocostatum* exposed to 0 (black solid line), 10 µ M (solid blue and orange lines) and 25 µ M (dashed lines) of Cu and Cd. Final cell density in all experimental conditions is annotated near the correspondent curve.

The exposure of the diatom *S. pseudocostaum* to two different concentrations of Cu and Cd (10 and 25 µ M) determined a decrease of cell density, that was found to be dose- and metal- dependent, with Cu-treated cultures showing a higher reduction of cell concentrations for the same metal dose administered. When exposed to 10 µ M of both heavy metals, cultures were still able to sustain a positive growth, even if their concentration after 72 hours of metal exposure were lower than that of the control. More specifically, final cell densities were 64% and 78% than that of metal-free cultures in Cu- and Cd- treated samples, respectively. Conversely, the higher dose (25 µ M) caused a strong inhibition of cell growth, with a final cell density of ca. 4% and 11% in Cu- and Cd- treated cultures with respect to that found in the control, respectively. Growth curves are shown in Fig 1, where we reported also the final concentrations, expressed as cells per ml of culture, for all experimental conditions.

Apart from the overall reduction in cell number in metal-exposed cultures, observations at optical microscope were not able to reveal specific damages to algal cultures at the lowest dose of Cu and Cd. Regarding the “harshest” conditions (e.g., exposure to 25 µ M of Cu and Cd), the only possible difference to highlight is the formation of “clusters”, probably due to the trapping of cells in exopolysaccharide matrixes after the death and the cytoplasm extrusion of unhealthy diatoms.

### Holographic tomography results

**Fig 2:**
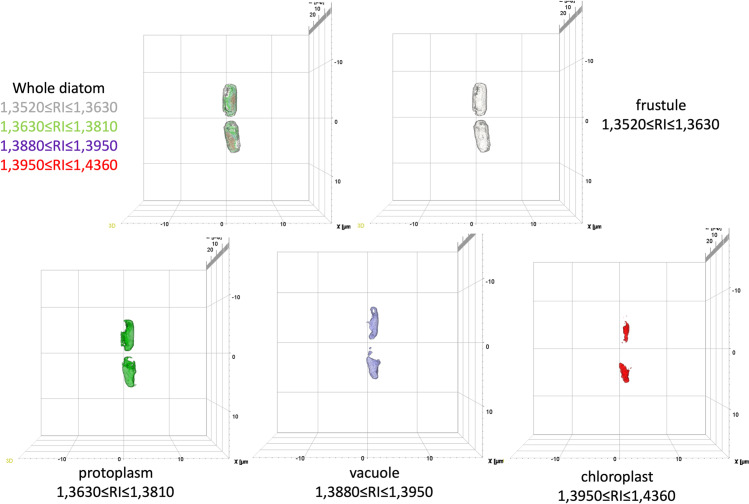
The subparts of the *S. pseudocostatum* highlighted by their RI values.

HT has been applied to image the 3D RI tomograms of *S.pseudocostatum* diatom in seawater. Starting from the tomogram, a quantitative analysis of the subcellular parts of the diatoms, i.e., frustules, chloroplasts, protoplasm and vacuoles, can be performed. To do this, the first step is to link the most appropriate RI range to each subcellular structure. By leveraging RI ranges adapted from Umemura *et al.* in 2020 [28] and assuming that the compositional structures of various diatom subparts are somewhat comparable across species, we set 1,3520 ≤ RI ≤ 1,3630 for the frustule, 1,3950 ≤ RI ≤ 1,4360 for the chloroplast regions, 1,360 ≤ RI ≤ 1,3810 for the protoplasm and 1,3880 ≤ RI ≤ 1,3950 for the vacuole. The RI tomogram of a healthy diatom is shown in Fig 2 as an example (see also S1 Movie). In particular, the above-mentioned subcellular structures corresponding to different RI ranges are reported with different colors. The same RI ranges have been applied to all the diatoms examined (both control and treated with heavy metals) and RI tomograms of diatom exposed to heavy metals at different concentrations for three days (T3) are also reported in S2 Fig.

#### Preliminary acquisitions.

**Fig 3:**
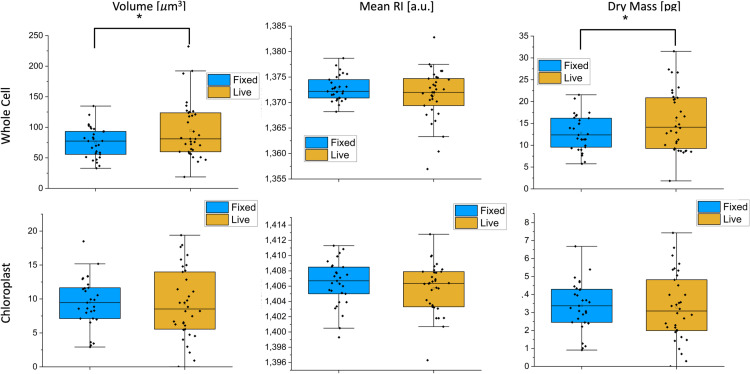
Bar plot for Volume, mean RI and dry mass for live and freshly fixed diatoms. First row: data related to whole cell; second row: data related to chloroplast. Graphs show the median (central horizontal line), standard deviations (box), and minimum and maximum values (vertical lines). T-test was performed for statistical significance: *0.01 < *p* ≤ 0.05.

Before initiating the main experiments, preliminary tests were conducted to ensure the fixation process did not alter the diatom cells. Both live and freshly fixed diatoms were imaged. Thirty cells were acquired for each condition, and parameters such as volume (µm^3^), mean RI (a.u.), dry mass (pg) and concentration (pg/ µm^3^) were assessed for both the whole cell and the sole chloroplast region. Fig 3 demonstrates that there were no significant variations between live and fixed diatoms, establishing the reliability of the fixation process.

This means that samples can be analyzed at any moment, and this feature is important when samples have to be collected at different time intervals. Moreover, sample fixation could be a useful tool also for natural samples (e.g., algal cells collected by polluted environments), since it avoids - during their transfer in the lab - eventual changes of cell physiological state which could be independent from the exposure to the toxic agent(s).

#### Control sample analysis.

**Fig 4:**
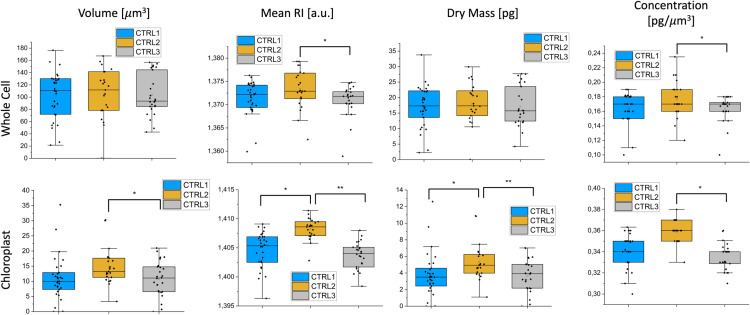
Bar plot for Volume, mean RI, dry mass and concentration for control sample triplicate at time T3. First row: data related to whole cell; second row: data related to chloroplast. Graphs show the median (central horizontal line), standard deviations (box), and minimum and maximum values (vertical lines). T-test was performed for statistical significance: *0.01 < *p* ≤ 0.05; **p ≤ 0.01.

A control sample triplicate at time T3 was analyzed to confirm the consistency among the triplicates. No significant differences were observed between CTRL1, CTRL2 and CTRL3, both in the chloroplast region and the whole cell (see Fig 4). Thirty cells were acquired for each control sample triplicate.

#### Data collection and analysis.

Considering these preliminary tests, a complete analysis on one member of the triplicate has been performed and CTRL, Cu 10μM, Cd 10μM, Cd 25μM and Cu 25μM at times T0, T1, T2 and T3 were acquired and analyzed. Thirty cells were acquired for each condition.

**Fig 5:**
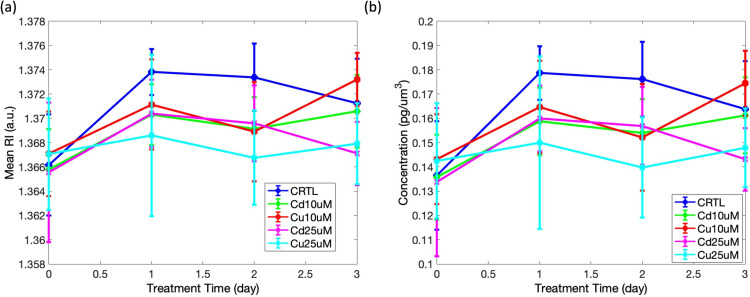
Plot of mean RI and mean concentration at different times and for different treatments. Data are referred to the whole cell.

The volume, mean RI, dry mass and concentration of the whole cell as well as of the chloroplast were measured and compared across different treatments and time points. Since chloroplast seems not to suffer the heavy metal exposure (see S2 Fig), here we report data retrieved for the whole cell. As a noteworthy result of the analysis we carried out, significant differences in mean RI and concentration were observed over time for each treatment group (CTRL, Cd 10 µ M, Cd 25 µ M, Cu 10 µ M, Cu 25 µ M). Thus, only the plots of these two metrics are reported here (see Fig 5) since they are found to be the most effective in describing the stress-induced variations in the sub-cellular distribution of the samples under test. The temporal changes for the mean RI and for concentration of whole cell are summarized in the plots in Fig 5(a) and 5(b) respectively, highlighting the dynamics of diatom responses to heavy metal exposure.

Additionally, the concentration of the diatom region obtained excluding the chloroplast (in the following this region is called chloroplast complement) was analyzed, and the absolute RI difference between the whole cell and the chloroplast was evaluated (we called this parameter “delta Mean RI”). The reason behind this choice is that chloroplast did not show a significant monotonic trend of the main measured parameters with the heavy metal doses (see S3 Fig). This result suggested us that excluding the chloroplast region could enhance the system sensitivity in detecting variations occurring as a function of the dose or the exposure time.

**Fig 6:**
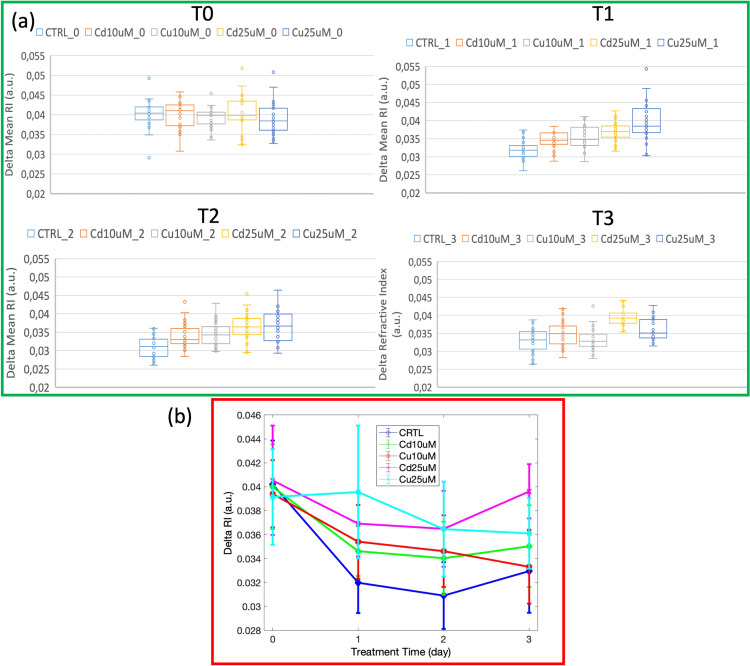
(a) Bar plot for delta mean RI at different times and for different doses. Graphs show the median (central horizontal line), standard deviations (box), and minimum and maximum values (vertical lines). (b) plot of mean values of delta mean RI at different times and for different doses.

Results are reported as boxplots in Fig 6(a) at a given time for each exposure; moreover, results of all the groups of examined samples at all considered times are summarized on the same plot, as showed in Fig 6(b).

**Fig 7:**
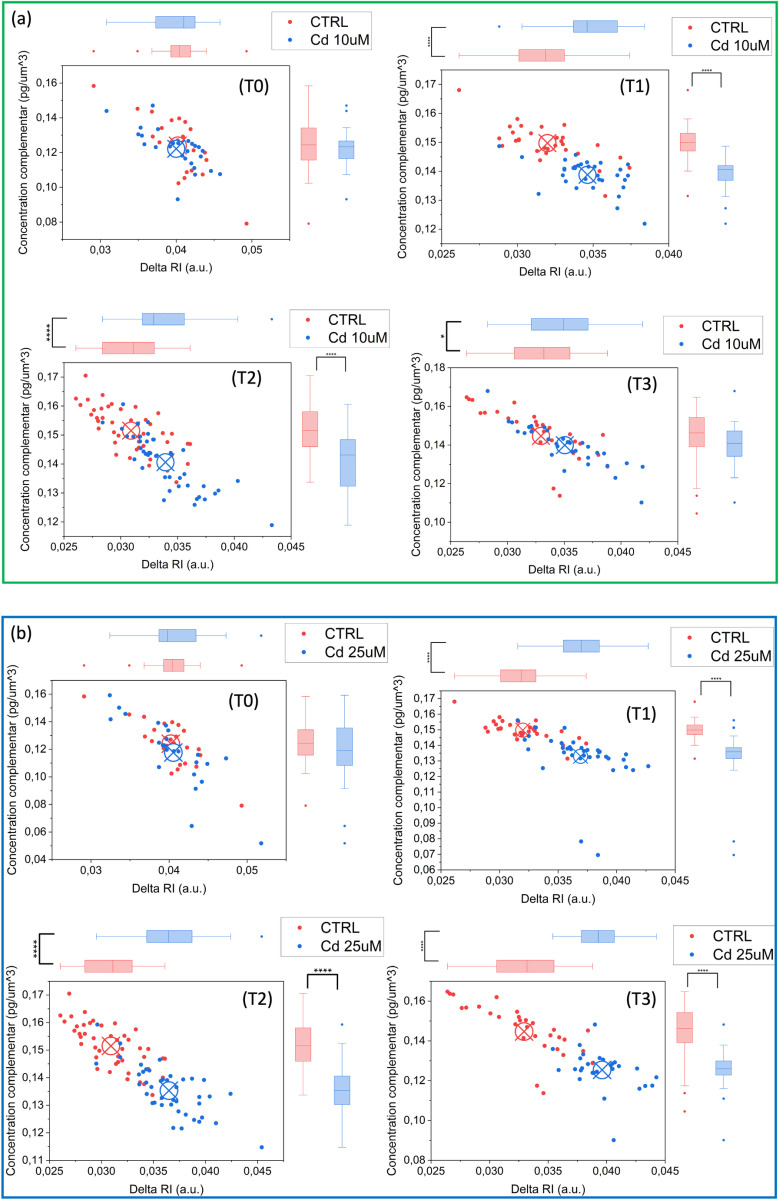
Cluster plot for different doses and exposure times: (a) Cd 10 µ M and (b) Cd 25 µ M. T-test was performed for statistical significance: *0.01 < *p* ≤ 0.05; **p ≤ 0.01; ***p ≤ 0.001; ****p ≤ 0.0001*.*

**Fig 8:**
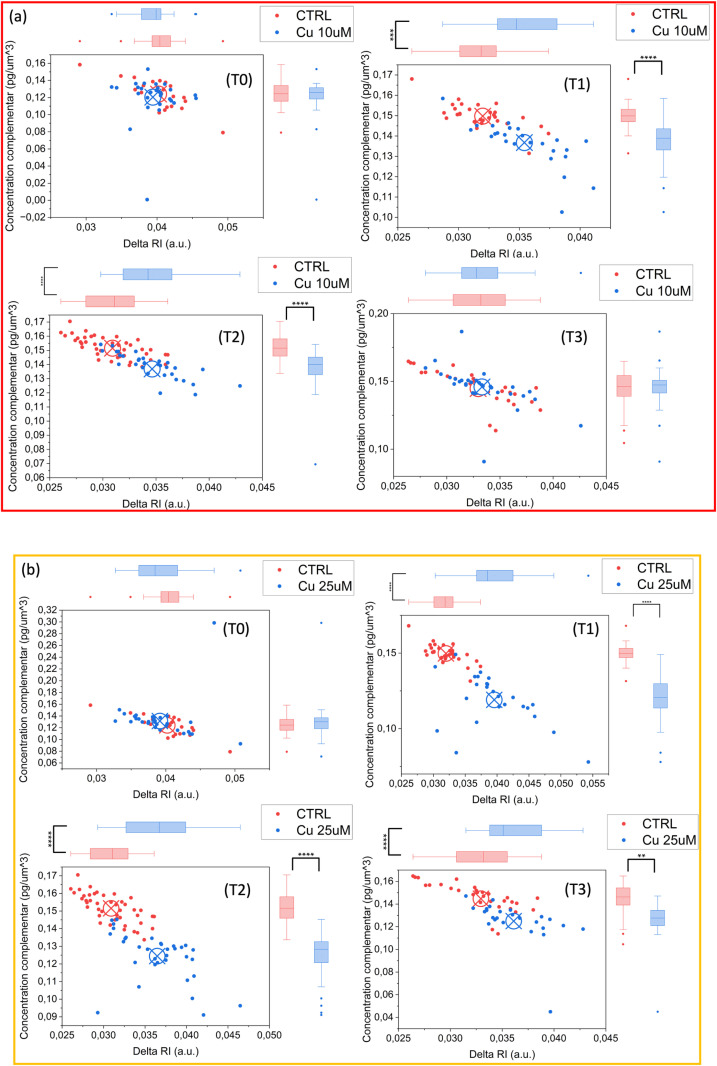
Cluster plot for different doses and exposure times: (a) Cu 10 µ M and (b) Cu 25 µ M. T-test was performed for statistical significance: *0.01 < *p* ≤ 0.05; **p ≤ 0.01; ***p ≤ 0.001; ****p ≤ 0.0001*.*

Statistical analyses using t-tests were performed at each time point (T0, T1, T2, T3) to compare parameters across all treatment groups. 2D scatterplots of the two main parameters (absolute value of RI difference between the mean RI obtained in the whole cell and the mean RI obtained in the chloroplast and concentration over the chloroplast complement) for all different doses and treatment have been measured and reported in Figs 7 and 8. This analysis revealed that at lower doses (10 µ M) of both Cd and Cu treatments, (Figs. 7(a) and 8(a)) data clusters appeared more distinct at T1 and T2 (*p* ≤ 0.0001), while they mixed with CTRL data at T3 (*p* > 0.05, not significant), suggesting a potential adaptation mechanism. At higher doses (25 µ M), Cd data showed clear differentiation starting from time T1, with highly significant differences compared to CTRL (*p* ≤ 0.0001 at T1, T2 and T3) (Fig. 7(b)). Conversely, Cu-treated samples remained significantly distinct from CTRL at T1 and T2 (*p* ≤ 0.0001), but at T3, the statistical significance was slightly lower but still notable (*p* ≤ 0.01), suggesting a possible higher tolerance or adaptability of *S. pseudocostatum* to Cu exposure (Fig 8(b)). The results confirmed that, unlike the chloroplast which does not significantly undergo heavy metal stress-induced alterations, the complementary regions showed notable changes.

The possibility to use only two parameters to differentiate between the doses in the sub-lethal doses region and for relatively short exposure times (e.g., T1), without relying on data-driven AI, is a more than welcome further indication of the potential use of *S. pseudocostatum* as a biosensor for Cu and Cd.

**Fig 9:**
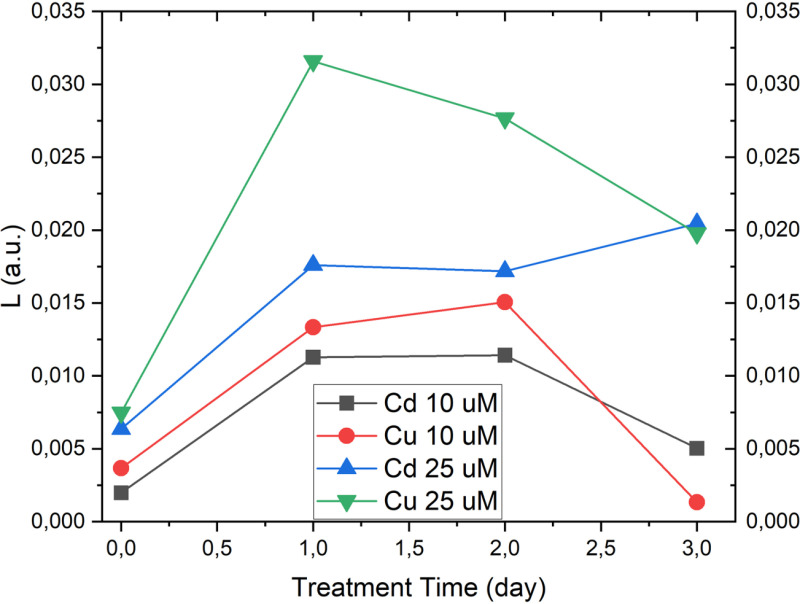
Centroids of each cluster showing the distances between the data clusters of CTRL and the four different dose/HM treatments at corresponding treatment times.

Centroid for each cluster have been evaluated. Distances between data cluster of CTRL at a given time and the four different dose/HM treatments at the same treatment time have been evaluated. Results, reported in the plot in Fig 9 further corroborated the findings, showing distinct clustering at lower doses and times T1 and T2 for all doses and HM, with mixing observed at T3 for lower doses, and clear differentiation for higher heavy metals concentrations. In detail, at higher doses (25μM), Cd data showed clear differentiation from T1, whereas Cu data remained distinct respect to CTRL samples at T1 and T2 but started to blend with CTRL data at T3, indicating a possible higher tolerance or adaptability to Cu. It is also evident that this blending effect for higher doses is less pronounced for the 25μM doses of both Cd and Cu, suggesting a lower capability of adaptation of the microalgae in the presence of higher concentration of heavy metals.

## Discussion

RI and internal concentration varied over time in samples, including metal-free ones. This could be due to natural biochemical variations in microalgal cells. Indeed, percentages of the primary metabolites (proteins, lipids and carbohydrates, which have different optical properties) may vary between the exponential and the stationary phase. In our experiments, we used an inoculum of healthy exponentially growing cultures of ca. 60’000 cells ml^-1^, and the cells reached the stationary phase after 72 hours. RI and concentration values showed the same trend as their values decreased at increasing metal concentrations.

Values were similar for control and samples exposed to 10 μM of both metals. In these samples, cells appear healthy also at optical microscope (Axiophot Zeiss, 200), and cells exposed to Cu and Cd are able, at these concentrations, to sustain a positive growth, that is only slightly lower than the control. Metals have a higher RI than cell components, so a high metal internalisation should increase the RI. Our results, instead, show that cells exposed to higher Cu or Cd concentrations (25 μM) have a lower RI and internal cell density. The most likely explanation of our results is that the “higher weigh” of metals adsorbed or internalized by microalgae is not sufficient to counteract the “loss of weight” due to cytoplasm extrusion or vacuolization, usually occurring under heavy-metal driven stress. Moreover, the brief time of exposure (72 hours) to these toxicants did not ensure the total removal of metals from the surrounding medium. In previous works, for example, we demonstrated that the species *S. pseudocostatum* removes ca. 14% of Cu when exposed to a culture medium amended with 10 µ M of this metal [42]. However, heavy metal removal is generally partial even with longer exposure times and varies among species and metals [43].

At high concentrations, Cu caused cell damages earlier than Cd (values of RI, concentration and Sphericity were lower than those of Cd-treated cultures after 24 and 48 h of exposure). Regarding the cell concentration measured over the chloroplast complement region, data show that this parameter decreases at increasing metal concentration. We can hypothesize that, since algae are affected by metal pollution in a dose-dependent manner, cell damages, including cytoplasm extrusion, is higher in samples exposed to 25 µ M of Cu and Cd and lower in samples able to sustain a positive growth (i.e., cultures exposed to sublethal doses, such as 10 µ M of Cu and Cd). Obviously, control cultures show the highest values of cell density, since no damages occurred during growth.

In control cultures and in samples exposed to sublethal doses of the toxicant, the internal cell density shows a coherent trend with time: it increases up to T2 and slightly decreases at T3. Time 3 coincides with the beginning of the stationary phase, when duplication rate is equal to mortality rate. Probably, at this time cells are “less healthy” and some of them can present damages which are independent on the exposure to toxic agents. In future work, further analyses with larger populations will be carried out to confirm or discard this hypothesis.

Despite the fact that the tested concentrations of Cu and Cd (i.e., 10 µ M and 25 µ M) are higher than typical environmental levels, they are comparable to those found in heavily contaminated areas in case of acute pollution, such as industrial discharge zones and mining-impacted waters, where metal contamination can reach similar levels.

Summing up, we can state that:

1. The reduction of average cell concentration is likely associated to their state of health. Indeed, chloroplast is not immediately damaged, but phenomena of cytoplasm extrusion and damages to other organelles contribute to a reduction of the average cell density. A dose-dependent effect has been observed, since this parameter is lower in cell exposed to lethal doses, and vice-versa. Further investigation, mainly focused on the assessment of eventual changes in the protoplasm region, and vacuoles size and number, are mandatory to understand if *S. pseudocostatum* undergoes vacuolization in response to heavy metal stress. This phenomenon has been already described in other diatoms [44] and can cause a partial “emptying” of the cells, thus contributing to the reduction of both mean RI and concentration, especially at high heavy metal doses.
2. These are preliminary results obtained by analysing a limited set of cells for each replicate of the 5 experimental conditions. To reduce statistical errors, a higher number of cells could be examined. In future works, in-flow phase contrast holographic tomography could be adopted to corroborate further these results by exploiting the high-throughput nature of this method [30]. However, the reported analysis is one of the first studies aimed at observing damages in HM-treated cultures, and we have been able to confirm a dose-dependent response of cells to Cd and Cu. To detect cell damages, this method is more rapid to other kinds of analyses, such as molecular and enzymatic assays. Moreover, the method is sensitive to sub-lethal doses at low exposure times, which is promising to setup lab protocols for detecting the heavy metal doses diatoms are exposed to.
3. With respect to our previous work on *S. pseudocostatum* used as bioprobe for Cu doses from 2D phase-contrast maps [3], the use of holographic tomography brings the advantage of inspecting the inner subcellular components of the diatoms and to link the stress-induced variations to the different sub-compartments. This approach can be complementary to the previously reported one, offering a deeper comprehension of the studied effects although linkable to a smaller number of samples.
4. Chloroplasts damages are not evident in the set of experiments performed in this work. In the future, we will add to the analysis experiments at lower cell density, e.g., with a ten-fold factor of density reduction. In this way, the amount of metal adsorbed/compartmentalised could increase, and we expect to find more damages in treated cultures, including those to chloroplasts.

Although further research is necessary to better elucidate the dose-dependent response of *S. pseudocostatum* to Cd and Cu, it should be noted that HT can be used as a complementary tool to conventional methods (enzymatic assays, molecular analysis, growth rate reduction assessment), since it allows a fast detection of eventual algal signs of stress. The main limit of the present technique is that *a priori* knowledge of the stressors is necessary, since it is able to determine eventual damages, but not the cause (in this case, the heavy metal exposure) of the side effects. On the other hand, the applicability domain of HT can be extended also to assess the effects of other kinds of stress (other metals, different pollutants, pH, salinity and temperature variations) which can cause detrimental effects to diatom species. Hence, HT can be used to observe side-effects also in the natural environments, by comparing eventual abnormalities in diatom shape in contaminated sites with respect to reference unpolluted ones. Moreover, considering that, in some cases, diatoms are able to incorporate some elements in their silica frustules without apparent side effects, HT could be also considered as a useful tool for the study of composite materials.

Indeed, differently from Cu and Cd, that are usually adsorbed onto the organic case of the diatoms and/or stored into their cytoplasm/organelles [45–48], previous works showed that diatoms can entrap Titanium and Platinum in their silica coating [49–51] without apparent detrimental effects, paving the way for development of novel, frustule-based electronic devices and photocatalysts. In light of this, we can assume that the use of HT (able to detect RI variations caused by metal entrapment), combined with other, validated techniques (observations at electron and optical microscopes, analysis of diatom elemental composition) has a dual function: it can be considered a novel method for a fast detection of sign of stress in diatoms, but can also be considered an investigative technique to study composite materials obtained by the natural embedding of trace elements in the silica casing for biotechnological applications.

## Conclusions

In conclusion, the research presented in this paper has provided compelling evidence that the diatom species *S. pseudocostatum* could serve as reliable bioindicator of metal pollution in water. One of the key advantages of using diatoms as indicators is their widespread distribution and abundance in virtually all freshwater and marine environments. This ubiquity allows for the development of comprehensive monitoring programs that can be tailored to specific geographical regions and their unique environmental challenges.

The study provides comprehensive insights into the responses of diatom subcellular parts to exposure. Through a complete analysis of HT studies, we have demonstrated the impact of Cd and Cu exposure on *S. pseudocostatum* subcellular components. The distinct changes in mean RI and concentration suggest that this diatom exhibit differential adaptability to Cd and Cu, with potential implications for understanding their survival mechanisms in polluted environments. The findings highlight that HT can be a useful tool for a fast detection of heavy-metal driven side-effects on *S. pseudocostatum*, even if complementary analyses are mandatory to better elucidate the mechanisms of “interaction” of Cd and Cu with the tested species, as well as the main physiological mechanisms which allowed the observed adaptation to metals over time.

## Supporting information

S1 Movie Multi-View 3D Visualization of *S. pseudocostatum.* Multi-view visualization of the diatom reported in Fig. 2, showing the rotation of the 3D image and its projection in the three planes (XY, YZ, and XZ) for enhanced spatial analysis. The subparts of the *S. pseudocostatum* are highlighted by their RI values reported in different colors.
(MP4)

S2 Fig The subparts of the *S. pseudocostatum* highlighted by their RI values: (a) Cd 10 µ M, (b) Cd 25 µ M, (c) Cu 10 µ M, (d) Cu 25 µ M. (PNG)

S3 Fig Plot of (a) mean RI and (b) mean concentration at different times and for different treatments. Data are referred to the chloroplast region.
(PNG)

S1 Data This file contains the raw data supporting Figures 1, 5, and 6 of the main manuscript.
(XLSX)
