# Supplementary figures and images for: Holographic tomography of the diatom Skeletonema pseudocostatum used as a bioindicator of heavy metal-polluted waters
Source: PLoS One. 2025 May 8;20(5):e0322960. doi: 10.1371/journal.pone.0322960 (PMC12061135; doi:10.1371/journal.pone.0322960)

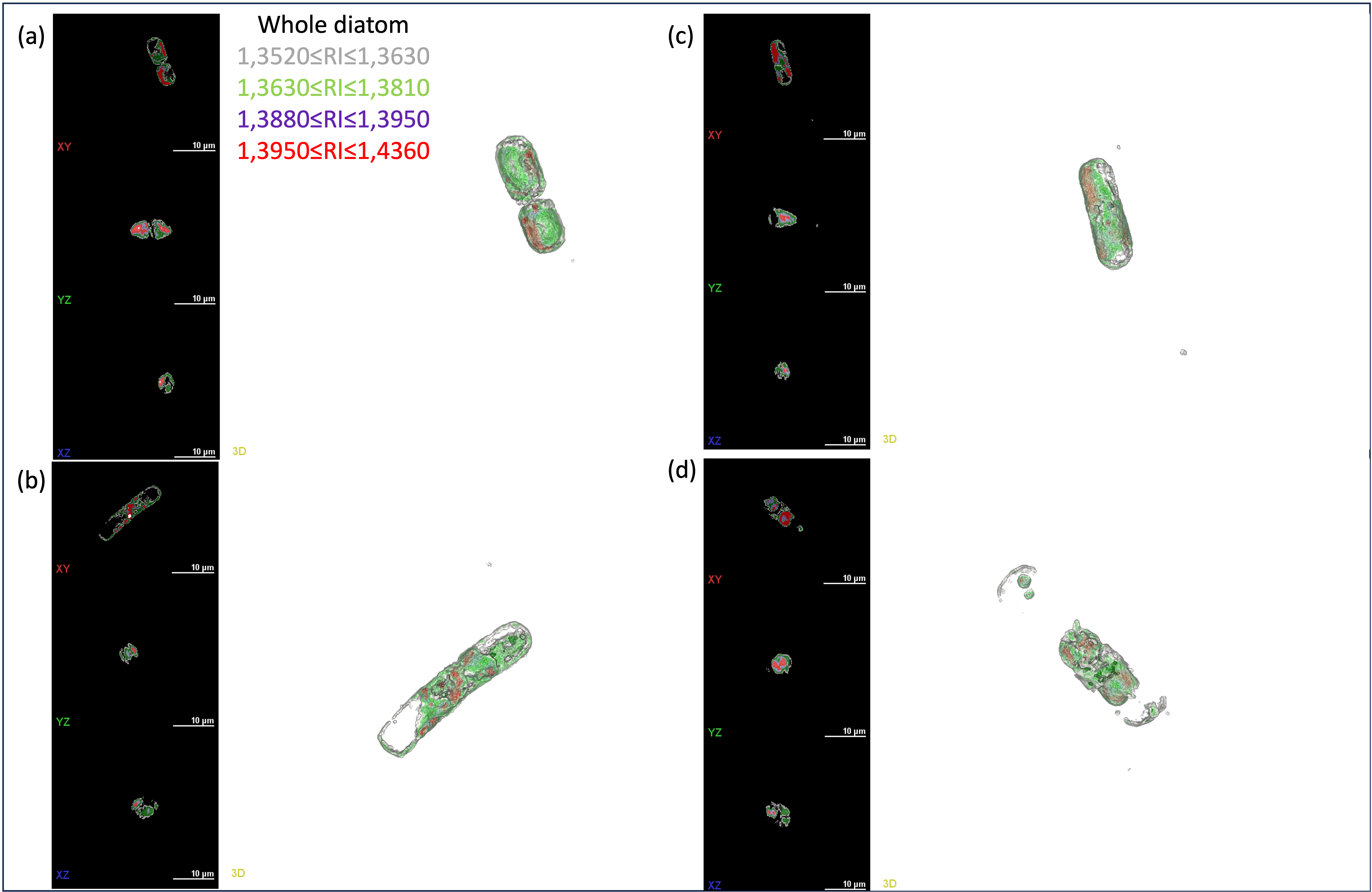

Supplement: S2 Fig — (PNG) [file pone.0322960.s002.png]

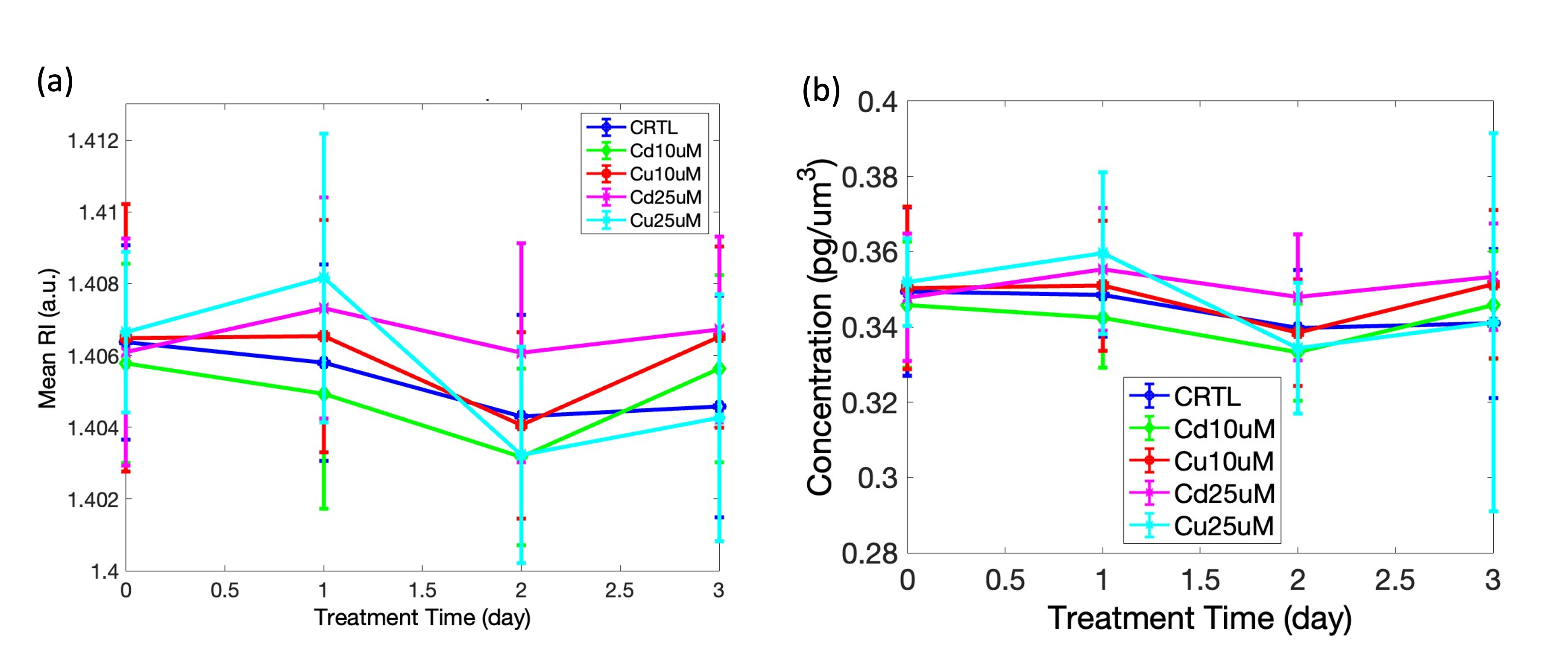

Supplement: S3 Fig — Data are referred to the chloroplast region. (PNG) [file pone.0322960.s003.png]
